# 

**Published:** 1894-10-27

**Authors:** 


**Notices of Births, Deaths, and Marriages** are inserted in this column at a charge of 2s. 6d. for an announcement not exceeding four lines.

**Notice to Advertisers and Subscribers.**

All Advertisements, Orders for Copies of The Hospital, and Business Communications should be addressed to **The Publisher, NOT TO THE EDITOR,** The Hospital, 428, Strand, W.C.

The Cost of Subscription, post free, is as follows:—Per week, 2½d.; per quarter, 2s. 8d.; per half-year, 5s. 3d.; per annum, 10s. 6d. Foreign:—Per Annum, 12s. 8d.; payable, in all cases, in advance.

### NOTICE TO CORRESPONDENTS.

All MS., Letters, Books for Review, and other matters intended for the Editor should be addressed The Editor, The Lodge, Porchester Square, London, W.

The Editor cannot undertake to return rejected MS., even when accompanied by stamped directed envelope.

**"Burdett's Hospital Annual," 1894.**

Fifth year of publication. 900 pp. Boards, gilt lettering. A most complete guide to British, Colonial, Indian, and American Hospitals, Dispensaries, Nursing and Convalescent Institutions, and Asylums. Price 5s. Published by The Scientific Press (Limited), 428, Strand, W.C.

**NOTICE.—The Index for Vol. XVI. (ending September 24th, 1894) is on sale at the Office of this Journal, Price 2½d., post free.**

### Scraps and Gleanings.

We learn that the Craig Epileptic Colony will not be ready to receive inmates for a period of probably three years.

Dr. D. G. Thomson, of Greenhill Gardens, Edinburgh, has left a bequest of £500 to the Edinburgh Royal Infirmary.

Lincoln County Hospital will benefit to the extent of about £6,000 through the bequest of the late Miss Anne Wells, of Leashingham.

It is finally decided in Lower Austria that a station for vaccinating against rabies shall be built somewhere in the neighbourhood of Vienna.

Baron Ferdinand de Rothschild, M.P., has, we learn, forwarded twenty guineas towards the £2,000 required by the Royal Chest Hospital in the City Road.

The fund for building the new infirmary immediately in the neighbourhood of Armagh on a site to be selected by a committee has now amounted to £2,600.

At a recent meeting of the Paddington Vestry, it was resolved that the vestry should approve of the erection of a small crematorium in its cemetery at Willesden.

Sir Thomas Wright has handed over £131 to the secretary of the Leicester Infirmary, an amount derived from the fête given by his kind permission in Abbey Park.

Much consternation is felt that the Clevedon Fever Hospital is proposed to be erected on Dial Hill, which hill is the popular recreation resort of the neighbourhood.

The annual report of the Cremation Society of England shows that a further success has crowned the work than in 1893. The total number of cremations during the year has been 131.

Addenbrooke's Hospital, Cambridge, has not been very rich in legacies this last year. Only one is mentioned in the annual report as having been received, and that is one of £45.

Mr. Hugh C. Smith, of London, has presented the land for the new accident hospital at Fleetwood, and towards the cost, which is calculated to be about £2,250, the same benefactor subscribed £300.

A German contemporary states that over £10,000 a-year is spent by the State in supporting laboratories in connection with the Berlin University, and the salaries of the Professors are not included in this.

The appeal made by the Bedford General Infirmary for funds for three additional cots in the children's ward is meeting with the response it deserves, though the necessary sum has not yet been reached.

The Secretary of the Meath Hospital and County Dublin Infirmary reports that he has received the sum of £108, the net proceeds of a sale of work held in the Town Hall, Bray, on behalf of the Convalescent Home of Bray.

The adoption of Hospital Saturday at Wrexham has done good service to the Wrexham Infirmary, and the pecuniary results have been so encouraging that it is hoped the event will become an annual fixture.

The thirty-third annual meeting of Directors of the Forfar Infirmary has just been held, the report of which states that the financial matters are satisfactory, and that a balance of £285 is left in favour of the Institution.

Great success has attended the first effort of the Leeds Ladies' Hospital Fund, and they are to be much congratulated on the result of their work. Something like £100 a month has been collected since the scheme was initiated in May last.

A cheque of £10 has been sent to the committee of Dr. Steeven's Hospital, Dublin, in recognition of the kindness shown to the families of married soldiers of the Dublin garrison. This was by command of Field-Marshal Viscount Wolseley.

The Buckingham Nursing Home celebrated its twenty-fifth anniversary on St. Luke's Day. This charity, which is entirely supported by voluntary contributions, is much in need of more substantial support than has so far been forthcoming.

The widow of Professor Billroth, the distinguished Austrian surgeon who died recently, has had a pension granted her by the Emperor of Austria which is three times larger than what has hitherto been customary to receive at the hands of the State.

To the local working men the Lincoln (County) Hospital has owed no less a contribution this year than nearly £700. This increase in the funds has come at an acceptable season, as serious expense has been incurred in repainting the whole of the exterior of the hospital.

During the last few months several improvements have been effected at the Rotherham Hospital and Dispensary. The wards have had all necessary appliances added for a system of thorough warming, and the drainage and sanitary matters have been carefully overhauled.

The increasing claims of cases for admission to the North Riding Infirmary have rendered it necessary to reopen the ward which had been closed for want of money to carry it on. This has imposed upon the committee of management the necessity of appealing to the public for increased subscriptions.

An American contemporary describes an excellent electric ambulance which has been planned to convey patients from the local dispensary (St. Louis) to the City Hospital. It is further intended to run the car to all parts of the city in response to ambulance calls, and the car itself is fitted up as an ambulance.

A long talked of Conference for the purpose of making arrangements for the provision of a small-pox joint hospital, to comprise Warrington Borough, the Rural Sanitary District, and the Leigh Joint Hospital District has just been held. The matter has been fully discussed, and is fast reaching a final settlement.

Baron Albert de Rothschild has contributed the munificent sum of half a million florins to the Vienna hospitals. This gift will be called the "Bettina Fund." The pavilion now in course of construction for female cancer patients on the Empress Elizabeth Hospital estate is an outcome of the Baron's liberality.

Owing to a large number of in-patients in the Stratford-on-Avon Infirmary the expenditure has this year exceeded the income by £122. The annual report further shows that there has been a falling off in some of the items of income, though two large bequests have been bequeathed to the hospital during the twelve months under review.

### Books Received.

Macmillan and Co.

"A Text-book of Pathology." By D. J. Hamilton, M.B., F.R.C.S.E.

Adam and Charles Black.

"The Senile Heart." By G. W. Balfour, M.D.

Walter Scott.

"The Cult of Beauty; a Handbook of Personal Hygiene." By J. C. S. Thompson.

"Apparitions and Thought Transference." By Frank Podmore, M.A.

T. Dobb, Liverpool.

"Notes on Nursing." Collected and Arranged by Nurse Evelyn Herbert.

J. and A. Churchill.

"Alpine Climates for Consumption." By Herbert Junius Hardwicke, M.D.

Sampson Low, Marston, and Co.

"Health and Condition in the Active and the Sedentary." By Nathaniel Edward Yorke-Davies.

Society for Promoting Christian Knowledge.

"Edible and Poisonous Mushrooms: What to Eat and What to Avoid." By M. C. Cooke, M.A., LL.D.

Homœopathic Publishing Company.

"Homœopathy: all about it; or the Principle of Cure." By John H. Clarke, M.D.

**Periodicals and Pamphlets.**—Guy's Hospital Gazette, Cassell's Family Magazine, St. Nicholas, The Island of Madeira (reprinted from the *Lancet*), Light in the Heart, Gustavus Adolphus, Our Little Dots, The Child's Companion, The Cottager and Artizan, Friendly Greetings, Revue Genéralé des Sciences, The Charity Organization Review, The Rural World.

### The Student of Medicine.

### APPOINTMENTS.

Dr. R. R. Allen, appointed Medical Officer of the Workhouse of the Dartford Union. Mr. J. C. H. Beaumont, appointed Medical Officer of the Infirmary and Workhouse of the Lewisham Union. Mr. E. W. Bliss, appointed Assistant Medical Officer to the Infirmary of the Parish of St. Marylebone. J. Busfield, M.R.C.S., L.R.C.P., F.I.C., appointed Medical Officer to Clapham General Dispensary. A. H. Churchill, M.R.C.S.Eng., L.S.A., appointed Consulting Medical Officer to the Walton-on-the-Hill Local Board. H. Collier, M.D., appointed Medical Officer of the Workhouse of the Great Yarmouth Union. A. E. Cope, M.B.Lond., B.S., appointed Medical Officer for the Third District of the St. George's Union. C. R. Crawford, M.R.C.S., L.R.C.P., appointed House-Physician to the Sussex County Hospital. H. Crutchley, M.D.Brux., L.R.C.P.Lond., L.F.P.S.Glasg., appointed Medical Officer of Health to the Alsagar Urban Sanitary District. T. A. Duke, appointed Medical Officer of the Third District of the Croydon Union. A. J. Fleming, M.D.R.U.I., L.R.C.S.I., appointed Medical Officer of the Workhouse of the Church Stretton Union. Mr. J. H. Godson, appointed Medical Officer of Health to the Cheadle and Gatley Urban Sanitary District. Q. McLennan, M.B., Ch.M., appointed one of the Surgeons-in-Chief to the Glasgow Royal Infirmary. J. M. Moir, M.D.Aberd., M.B., C.M., appointed Honorary Physician to the Northern Counties Institute for the Blind, Inverness. Mr. P. J. Probyn, appointed Assistant Medical Officer of the Workhouse and Infirmary of the Parish of St. Leonard, Shoreditch. Dr. J. Proctor, appointed Medical Officer for the Effingham District of the Dorking Union. R. F. Shaw, L.R.C.P., L.R.C.S.Edin., appointed Medical Officer of Health to the Whitley Upper Urban Sanitary District. Mr. W. A. Shilliton, appointed Medical Officer for the Sixteenth District of the North Bierley Union. W. K. Sibley, M.A., M.D., B.C., M.R.C.P., appointed Clinical Assistant at the Chelsea Hospital for Women. J. P. Stallard, M.B.Edin., appointed Physician to Manchester Southern Hospital for Diseases of Women and Children. Mr. C. Stuart, appointed Medical Officer for the Tenth District of the North Bierley Union. A. F. Walker, L.R.C.P., L.R.C.S.Edin., appointed Senior Assistant Medical Officer at the Infirmary of the Township of Toxteth Park. H. Watson, M.D.Aberd., reappointed Medical Officer for the Fifth District of the Norwich Union. N. P. Watt, M.A., M.B.Edin., appointed Assistant Resident Medical Officer to the Royal Lunatic Asylum, Dundee. E. C. Williams, appointed Medical Officer of Health to the Presteigne Urban Sanitary District. C. Wilson, M.B., C.M.Glasg., appointed Resident Medical Officer to the Victoria Hospital. J. H. Collier, M.B., C.M.Aber., L.R.C.P., L.R.C.S.Ed., L.F.P. and S.G., has been appointed Medical Resident Officer to the Sheffield Union Hospital.

### VACANCIES.

#### Resident Medical Officers.

Boscombe Hospital, Shelley Road, Boscombe, Bournemouth.—House Surgeon; unmarried. Salary £60 per annum, with board and residence. Applications to the Honorary Secretary by October 30.

Hospital for Women and Lying-in Institute, Brighton.—House Surgeon; unmarried, and under 30 years of age. Salary £80 per annum, with board and rooms. Applications to the Secretary by November 15th.

London Temperance Hospital, Hampstead Road, N.W.—Assistant Resident Medical Officer, doubly qualified. Appointment for six months. No salary, but board, washing, and residence provided, and an honorarium of 5 guineas on satisfactory completion of the term of appointment. Applications to E. Wilson Taylor, Secretary, by November 2nd.

Manchester Hospital for Consumption and Diseases of the Throat, Bowdon, Cheshire.—Residence Medical Officer. Salary £60 per annum, with board, apartments, and washing. Applications to C. W. Hunt, Secretary, by October 29th.

### THE STUDENT OF MEDICINE—*continued.*

North-West London Hospital, Kentish Town Road, N.W.—Resident Medical Officer, and Assistant Resident Medical Officer. Salary £50, attached to the senior post. Appointments for six months. Applications to Alfred Craske, Secretary, by October 29th.

Smedley's Hydropathic Establishment, Matlock.—House Physician. Must be under 30 years of age. Appointment for six months. Honorarium £40, with extension, if mutually desired, at the rate of £120 per annum. Board, lodging, &c., provided. Applications to the Secretary by October 30th.

#### Non-Resident Medical Officers.

Central London Ophthalmic Hospital, Gray's Inn Road, W.C.—Two Assistant Surgeons. Applications to the Secretary by November 5th.

Charing Cross Hospital.—Surgical Registrar. Must be qualified to practise. Salary £40 per annum. Applications to the Chairman, Medical Committee, by October 29th.

North London Hospital for Consumption, Hampstead.—Assistant Physician. Candidates must be graduated in Medicine of a University of the United Kingdom or Fellows or Members of the Royal College of Physicians of London. Applications to L. F. Hill, M.A., Secretary, at the Office, before November 3rd.

Royal Westminster Ophthalmic Hospital, King William Street, West Strand, W.C.—Two Assistant Surgeons. Applications to T. Beattie-Campbell, Secretary, by November 3rd.

Sunderland Infirmary.—Honorary Physician. Applications to the Chairman of the Medical Board by November 5th.

### Medical Appointments.

**NORTH LONDON HOSPITAL FOR CONSUMPTION, HAMPSTEAD.**

Offices and Out-patient Department, 41, Fitzroy Square, W.

ASSISTANT PHYSICIAN.

